# Elastographic Imaging of Anaplastic Seminoma of Testis With Its Ultrasound and Doppler Correlation: A Case Report

**DOI:** 10.7759/cureus.32813

**Published:** 2022-12-22

**Authors:** Pratik J Bhansali, Suresh V Phatak, Gaurav V Mishra, Vadlamudi Nagendra

**Affiliations:** 1 Department of Radiodiagnosis, Datta Meghe Institute of Medical Sciences, Wardha, IND

**Keywords:** testicular tumour, elastography, colour doppler, ultrasonography, anaplastic seminoma

## Abstract

The commonest solid tumour in men between the ages of 15 and 44 is testicular cancer. Germ cell tumours, which are then subdivided into seminomatous and non-seminomatous tumours, are its primary histological kind. In the fourth decade of a man's life, seminoma accounts for 55% of testicular cancer. Anaplastic seminoma, which accounts for 5% to 15% of testicular seminoma, is an uncommon kind of seminoma. The anaplastic variant of classical seminoma is an uncommon type of seminoma. In order to increase confidence in diagnosing and differentiating benign from malignant lesions and localising lesions in the testis, tissue elastography has arisen as a definite, important supplementary method. We present a case report of anaplastic seminoma with its classical imaging findings on strain elastography and its correlation with ultrasound and doppler.

## Introduction

Testicular seminomas are classified as spermatocytes, classical seminoma (CS) as well as anaplastic variations of CS. Histologically anaplastic seminoma ranges from 5% to 15% [1]. Histological diagnosis is done when there are few lymphocytes, pleomorphic cells having non-clear cytoplasm, focal necrosis, no fibrovascular septae, cellular irregularity and >3 mitotic figures per high power field [2]. The testis is usually assessed using B-mode ultrasonography and colour Doppler, with its excellent anatomical delineation and location of the testis which is superficial and has been a major mainstay for finding and characterising localised testicular lesions. Location whether extratesticular or intratesticular, the shape of the lesion, size of the lesion and its echogenicity pattern of lesions are all precisely determined by B-mode ultrasound. The vascularity of the testicular tissue can be useful in determining whether the lesion is benign or malignant. Colour Doppler evaluates the existence and vascularity pattern in and around the lesion to thrust diagnostic spirit. Assuming that the lesions which are harder are malignant than benign, advanced radiological techniques like tissue elastography have surfaced as an important tool in terms of quality and quantity adjunct to conventional tools to give extra details on the stiffness of the tissue [3]. These techniques aim to promote improvement in the diagnosis of malignant lesions and benign lesions.

## Case presentation

A 75 years old gentleman presented with a swelling on the right scrotum which was painless for one year and increased in size gradually. General and systemic examination was normal. On local examination of the right testis: a palpable mass was noted in the right side of the scrotum, which was firm, approximate size measuring 8 x 5 cm and getting above the swelling was possible. The fluctuation and transillumination tests were negative. Touch and pain sensations over the scrotal wall were present. The left side of the scrotum was normal.

**Figure 1:**
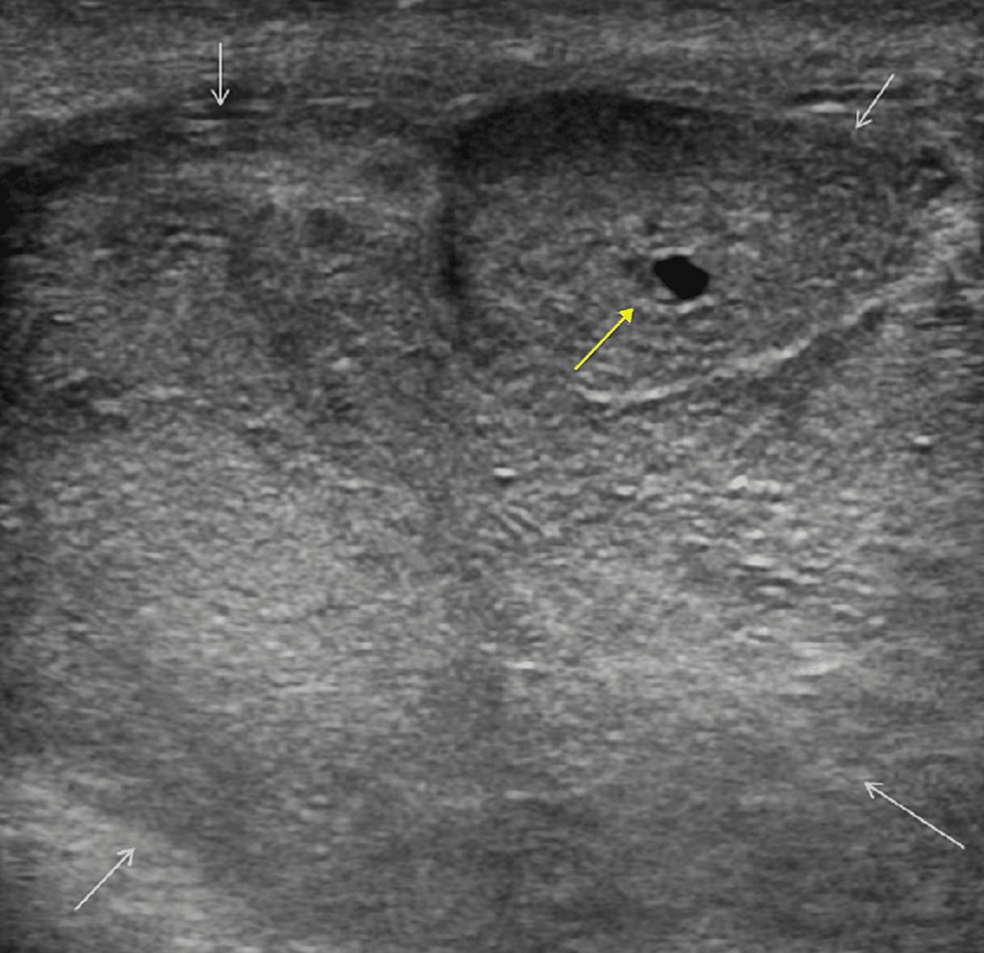
Ultrasound of right testis showing a mass lesion with heterogeneous echotexture including hypoechoic and hyperechoic areas (white arrow) and a small cystic area (yellow arrow).

**Figure 2:**
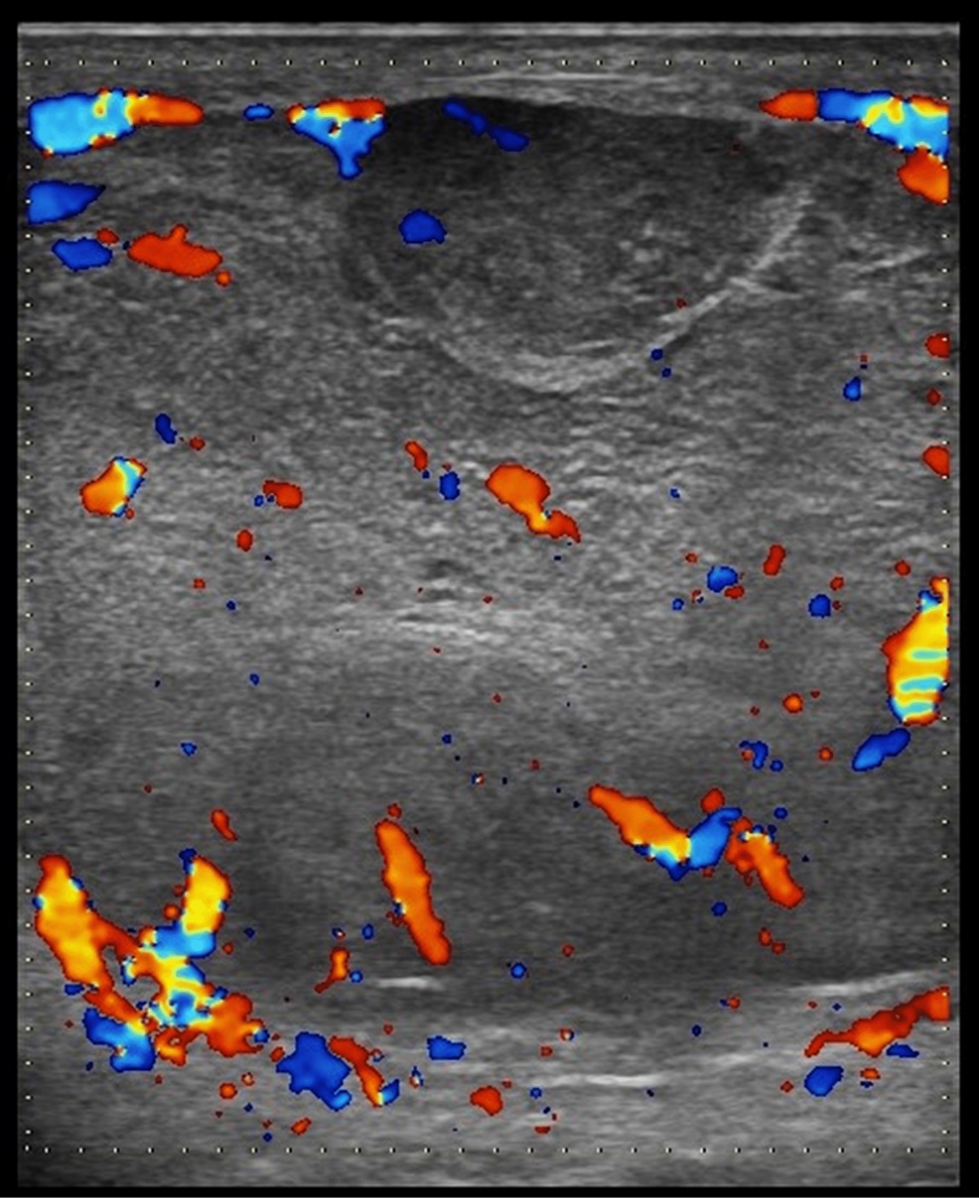
Doppler imaging of the right testis showing increased vascularity in the peripheral parts.

Ultrasonography of the scrotum revealed a mass showing a heterogenous echotexture. A small cystic area is also seen with increased vascularity on the doppler, as shown in Figures 1-2.

**Figure 3:**
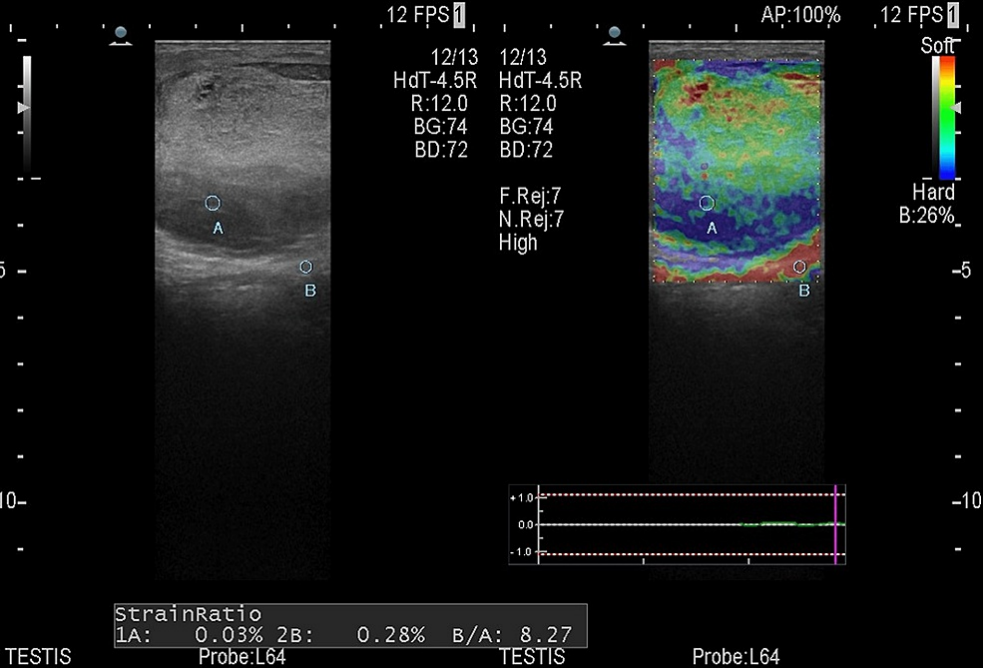
Strain elastography revealed a score of 5 on the visual elasticity score and a strain ratio of 8.2. A: Region of a lesion used for calculating strain ratio B: Region of a normal tissue used for calculating strain ratio

Strain elastography revealed a score of 5 on the visual elasticity score, and the strain ratio of the lesion was 8.2, as shown in Figure 3.

The value of tumour markers and beta-human chorionic gonadotropin (HCG) was 11.98 IU/L (normal range 0-2.5 IU/L). No abdominal lymphadenopathy was seen on the USG abdomen. These findings were suggestive of testicular malignancy. High inguinal orchidectomy was performed, and histopathology suggested it to be anaplastic seminoma stage IB. The patient was referred to the oncology section for chemotherapy. Chemotherapy consisted of cisplatin, etoposide and bleomycin regimen. After the completion of two cycles of chemotherapy, tumour markers were normal. On follow-up, the patient was doing well.

## Discussion

Assessing the testis with the help of B-mode along with colour Doppler ultrasonography is a favoured imaging examination to assess and make a diagnosis of testicular pathology as it gives exquisite anatomical details of the testicular anatomy. The shape, size, extent, whether intratesticular or extra testicular origin and determining various patterns of echogenicity of the lesion can be distinguished precisely on B-mode USG. Colour Doppler assists with vascularity and type of blood flow in the pathologic area, which aids in more precise findings. Newer and higher sonographic procedures, for example, tissue elastography, is of great help in qualitative, likewise, possibly quantitative information to give more data on tissue stiffness; with this data separating malignant from benign pathologies is considerably more straightforward and more accurate, under the consideration that benign lesion is softer as compared with the stiffness of malignant lesion which is harder [3].

Normal testis on strain elastography demonstrates a classical three-ring structure which is blue bordered surrounded by red bands and central green colour of parenchyma [4]. Lesions within the testis can be further classified as benign, non-neoplastic benign and malignant neoplasms. Among all primary malignant testicular tumours, more than 95% belong to testicular germ cell tumours and are further split into non-seminomatous germ cell tumours or seminomatous germ cell tumours, which form 60% and 40% of all germ cell tumours accordingly. Testicular seminomas have a specific character in that they are homogeneously hypoechoic lesions that are well-defined, showing no local invasion of the tunica albuginea on USG. On strain elastography features of seminomas present a consistently stiff nature throughout. Teratoma, embryonal cell carcinoma, mixed tumours and choriocarcinoma are categorised as non-seminomatous germ cell tumours containing two or more histological cell types. They usually also show rigid tissue stiffness and have less homogeneous elastographic features mainly because of heterogeneous cellularity. Five per cent of all testicular neoplasms are gonadal stromal tumours like Leydig cell tumours and Sertoli cell tumours. Most Leydig cell tumours are benign; nearly 10% of them show malignant potential on histology [5]. Gonadal stromal cell tumours more commonly show well-demarcated solitary testicular lesions, which are small in size with increased peripheral vascularity and reduced reflectivity on Doppler. On strain elastography, tissue stiffness is between mildly hard to hard [6,7]. Seminoma is the most commonly occurring pure germ cell tumour with an incidence of 35-50% amongst all germ cell tumours. Its incidence is predominantly in an older age group when compared to non-seminomatous tumours [8].

Strain elastography describes six scores in the form of visual elasticity scores shown in Table 1 [7].

**Table 1:** Visual elasticity scores based on strain elastography.

Visual elasticity score	Description of strain elastography
Score 1	Lesion is green with tiny red spots in between
Score 2	Homogeneously green colour
Score 3	Lesion is green with few blue spots in between
Score 4	Lesion is green in the periphery and blue in the centre
Score 5	Lesion is blue with central red and green spots
Score 6	Lesion is completely blue

Visual elasticity score is said to be hard (score >3) and soft (score <3). Hard lesions are seen in malignant tumours. Higher values of strain ratio are observed in malignant lesions [7].

Magnetic resonance imaging has been accurately differentiating between seminomatous and non-seminomatous testicular neoplasm. Seminomas are seen as low signal intensity on T2-weighted images with multinodular sharply defined homogenous intensity. In seminoma visualisation of fibrovascular septa, demonstrating low signal intensity on T2WI and enhancement more than background tumour on T1WI is one of the key features along with fibrovascular septa, which can be thin or thick showing variably low signal intensity and correlates with the fibrous capsule on histology [9]. Differential diagnosis can be extensive in the form of non-neoplastic and neoplastic lesions. There are various tumours which have similar features to that of testicular seminoma in imaging. Benign conditions such as sarcoidosis, splenogonadal fusion, epidermoid cyst, segmental infarction, adrenal rests and hematoma are also sex cord-stromal tumours. In contrast, malignant mimics are lymphomas, non-seminomatous germ-cell tumours and metastasis [10].

## Conclusions

Strain elastography has set a new level in ultrasound radiology. Further evaluation of tissue stiffness in the ultrasound is possible with strain elastography. As seen in this case, strain elastography helps in differentiating benign and malignant lesions, thus increasing diagnostic confidence along with a multiparametric evaluation based on ultrasonographic appearance, colour doppler and tissue elastography.
